# Sex differences in depressive symptoms, psychological distress and workplace discrimination among workers in Chilean large-scale mining: implications for human-centered ergonomics and sustainable work

**DOI:** 10.3389/fpubh.2026.1917074

**Published:** 2026-08-12

**Authors:** Gonzalo Bravo-Rojas, Héctor Ignacio Castellucci, Alejandra Fuentes-García, José Ignacio Méndez

**Affiliations:** 1 Programa de Doctorado en Salud Pública, Facultad de Medicina, Universidad de Chile, Santiago, Chile; 2 Centro de Estudio del Trabajo y Factores Humanos, Facultad de Medicina, Universidad de Valparaíso, Valparaíso, Chile; 3 Escuela de Salud Pública, Facultad de Medicina, Universidad de Chile, Santiago, Chile; 4 Departamento de Medicina Familiar, Facultad de Medicina, U. Católica de Chile, Santiago, Chile; 5 Programa Medicina del Trabajo y del Ambiente, U. San Sebastián, Valdivia, Chile

**Keywords:** depression, mental health, mining, occupational health, psychological distress, workplace discrimination

## Abstract

**Objectives:**

To examine sex-related differences in depressive symptoms, psychological distress, and perceived workplace discrimination among workers in a large-scale mining company in Chile, considering these outcomes as indicators of occupational wellbeing and sustainable work from a human-centered and organizational ergonomics perspective.

**Methods:**

A cross-sectional study was conducted between September and December 2024, including both direct employees and contractor workers from a large-scale mining company. Data were collected through an online questionnaire that included sociodemographic and occupational variables, the Patient Health Questionnaire-9 (PHQ-9), the Kessler Psychological Distress Scale (K6), and a discrimination questionnaire developed by the authors. Overall and sex-specific prevalence estimates were calculated. Crude and adjusted prevalence ratios (PRs) with 95% confidence intervals (95% CIs) were estimated using Poisson regression models with robust variance.

**Results:**

Among the 712 participants, 79.9% were men and 20.1% were women. The overall prevalence of depressive symptoms (PHQ-9 ≥10) was 8.3%, with a significantly higher prevalence among women (14.5%) than men (6.8%) (adjusted PR = 1.81; 95% CI: 1.01–3.25). Psychological distress (K6 ≥7) was reported by 15.2% of workers and was more prevalent among women (21.2%) than men (13.8%) (adjusted PR = 1.30; 95% (0.85–1.97). Perceived workplace discrimination was reported by 13.6% of participants and was also more common among women (18.5%) than men (12.4%) (adjusted PR = 1.67; 95% CI: 1.04–2.68).

**Conclusions:**

This study identified a higher prevalence of depressive symptoms, psychological distress, and perceived workplace discrimination among women compared with men working in the large-scale mining sector. However, only the differences in depressive symptoms and perceived workplace discrimination remained statistically significant after adjustment for sociodemographic and occupational factors.

## 1 Introduction

Mental health in the workplace has gained increasing attention within the field of occupational health, particularly in industries such as mining, which are characterized by demanding working conditions and intensive work arrangements such as the Fly-in/Fly-out (FIFO) model, in which workers commute from remote areas to mining sites (1, 2). In this context, there is growing recognition that organizational and psychosocial factors are essential components of occupational wellbeing and the long-term sustainability of work.

Although the FIFO system facilitates access to specialized labor, it also presents challenges for work–life balance (3) and has been associated with adverse physical and mental health outcomes, including stress, anxiety, and fatigue (1). Furthermore, it may generate tensions within host communities due to the limited economic benefits they perceive (2). Mining operations are continuous and are often organized through shift work systems. Night shifts, in particular, have been associated with sleep disturbances and cognitive impairment, increasing occupational risks and compromising workers' wellbeing (4).

At the organizational level, the mining industry faces significant cultural and structural challenges. A report by Rio Tinto documented substantial levels of workplace harassment, including sexual harassment and racism (5), highlighting the need to promote inclusive and respectful organizational cultures. Economic conditions, particularly fluctuations in international copper prices, also affect employment conditions. Evidence suggests that periods of rising copper prices are associated with increased operational pressure and a higher risk of fatal incidents, especially among direct employees (6). These challenges are relevant to achieving the Sustainable Development Goals, particularly SDG 8 on decent work and economic growth, as workplace conditions and labor relations can directly influence workers' health, inclusion, and wellbeing.

Within this context, women remain particularly vulnerable due to persistent gender inequalities. Mining has historically been a male-dominated industry, influenced by legislation such as the Mines and Collieries Act of 1842, which prohibited women from working underground. Although intended to protect health, this legislation institutionalized exclusion and reinforced patterns of hypermasculinity, relegating women to informal and precarious roles (7). These patterns continue to persist, as reflected in the low representation of women in the mining workforce (8).

In Chile, copper mining poses important occupational health challenges. Workers are exposed to both physical and psychological hazards, including silicosis, musculoskeletal disorders, and high levels of stress associated with excessive demands and extended working hours (9). Despite these risks, mental health remains a relatively underrecognized issue. A Chilean study conducted in 2010 reported depressive symptoms in 23% of mining workers, associated with high levels of stress, effort–reward imbalance, and low supervisory support (10). Despite generally positive self-rated health, these findings suggest that mental health concerns remain insufficiently visible within the sector (11). In response, Chile has established gender equity targets aimed at increasing women's participation in the mining workforce to 20% by 2030 and 35% by 2050, while also increasing women's representation in leadership positions to 25 and 40%, respectively (12). Understanding these inequalities is important for developing occupational health strategies that promote more inclusive, equitable, and sustainable work environments in the mining sector.

Against this background, understanding mental health and workplace discrimination is essential for advancing human-centered approaches to occupational health and sustainable work in the mining sector. From an organizational ergonomics perspective, psychosocial outcomes such as depressive symptoms, psychological distress, and workplace discrimination can be viewed as indicators of how work systems support workers' wellbeing, inclusion, and long-term participation in the workforce. Therefore, the objective of this study was to examine sex-related differences in depressive symptoms, psychological distress, and perceived workplace discrimination among workers employed by a large-scale mining company in Chile. By identifying potential inequalities in these outcomes, this study seeks to contribute evidence for the development of healthier, more inclusive, and sustainable mining workplaces, and to discuss their implications for occupational wellbeing and sustainable work in the mining sector.

## 2 Methods

### 2.1 Study design and sample

A cross-sectional study was conducted among workers from a large-scale mining company in Chile, including both direct employees of the principal company and workers employed by contractor companies.

### 2.2 Setting

The study was carried out in a mining company between September and December 2024. Participants were invited through two recruitment channels. Direct employees of the principal company received an invitation via their institutional email accounts. The invitation included general information about the study and a link to the online questionnaire hosted on the SurveyMonkey platform (13). Contractor workers received the same questionnaire link through their direct supervisors, who distributed it via WhatsApp or through email addresses registered with the contractor companies. Because the invitation of contractor workers was coordinated independently by multiple contractor companies and the total number of eligible contractor workers was not available, the exact number of contractor workers invited to participate could not be determined. Consequently, an overall response rate for the study could not be calculated. The questionnaire consisted of four sections: sociodemographic and occupational characteristics, depressive symptoms, psychological distress, and perceived workplace discrimination.

### 2.3 Participants

Eligible participants were active workers employed either by the large-scale mining company or by contractor companies providing services to the principal company during the data collection period. All participants provided informed consent through a digital consent form before accessing the survey, ensuring compliance with the ethical requirements of the study. Participation was voluntary, and the informed consent form explicitly stated that all information would be treated confidentially. Supervisors were responsible only for distributing the survey link and had no access to information regarding participation or individual responses. Participants were not required to provide any personal identifying information to complete the questionnaire. However, those who wished to receive a summary of the study findings could voluntarily provide an email address at the end of the survey. This information was collected separately from the survey responses, was used exclusively for this purpose, and was accessible only to the principal investigator. The participating mining company did not have, and will not have, access to individual responses, participation records, or identifying information.

### 2.4 Variables

The main outcomes of interest were depressive symptoms, psychological distress, and perceived workplace discrimination, assessed as follows:

### 2.5 Depressive symptoms

Depressive symptoms were assessed using the Patient Health Questionnaire-9 (PHQ-9), which has been previously validated in Chile (14). The instrument consists of nine items that assess the frequency of depressive symptoms during the previous two weeks. Responses are recorded on a four-point Likert scale ranging from 0 to 3 (0 = not at all, 1 = several days, 2 = more than half the days, and 3 = nearly every day). Total scores range from 0 to 27 and are interpreted as follows: 5–9 mild symptoms, 10–14 moderate symptoms, 15–19 moderately severe symptoms, and ≥20 severe symptoms. In this study, a cutoff score of ≥10 was used to classify participants as having moderate-to-severe depressive symptoms (≥10) vs. mild or no depressive symptoms (< 10), consistent with recommendations for identifying clinically significant depressive symptoms (15).

#### 2.5.1 Psychological distress

Psychological distress was assessed using the Kessler Psychological Distress Scale (K6) (16), a tool validated in Chile for measuring general psychological distress (17). The questionnaire includes six items evaluating the frequency of anxiety and depressive symptoms experienced during the previous month. Total scores range from 0 to 24 and are categorized into four levels: no distress = 0, moderate distress = 1–6, high distress = 7–12, and very high distress = 13–24. For prevalence estimation, a cutoff score of ≥7 was used, classifying participants with high or very high levels of distress as cases. This threshold has been used in previous studies to identify individuals at elevated risk of developing mental disorders (18, 19).

#### 2.5.2 Perceived workplace discrimination.

Perceived workplace discrimination was assessed using a questionnaire developed by the authors (20). The questionnaire consists of 14 primary items (plus two optional open-ended questions). The first section includes four items assessing the prevalence of perceived and observed workplace discrimination and the perceived reasons for these experiences. The second section comprises 10 items designed to characterize discriminatory events, including the perpetrator, the context in which discrimination occurred, the frequency and type of discrimination, and its perceived effects on health and work performance.

The questionnaire demonstrated adequate content validity through expert review and was developed as an exploratory instrument to describe and characterize workplace discrimination in the Chilean mining sector (20).

For the purposes of the present study, only the two items assessing the prevalence of perceived and observed workplace discrimination were used as study outcomes. For both items, workplace discrimination was defined as any unfair or negative treatment toward employees based on individual characteristics or social group membership that is not related to job performance. Perceived workplace discrimination was assessed with the question, “In the past year, have you felt discriminated against in your workplace?”, while observed workplace discrimination was assessed with the question, “In the past year, have you observed acts of discrimination against other people in your workplace?”. Both outcomes were measured as dichotomous variables (yes/no), and participants answering “yes” were classified as cases for the corresponding outcome. The remaining items were used descriptively to characterize reported discriminatory experiences.

### 2.6 Covariates

The following covariates were included to characterize the study population: age ( ≤ 29 years, 30–44 years, and ≥45 years), company type (principal company or contractor), job function (administrative, field-based, or both), shift system (shift work vs. non-shift work), tenure with the company (< 5 years, 5–10 years, 11–20 years, and >20 years), tenure in the mining sector (< 5 years, 5–10 years, 11–20 years, and >20 years), parental status (yes/no), marital status (single, married, civil union, separated, divorced, or widowed), educational attainment (primary/secondary education, technical education, university/postgraduate education), and awareness of the company's anti-discrimination policy (yes/no). Sex was considered the stratification variable.

### 2.7 Sample size

The sample size was estimated based on a finite population of 10,000 workers, assuming a 95% confidence level, a 5% margin of error, and an expected prevalence of 50%, resulting in a minimum required sample of 370 participants. Because of variability in the number of contractor workers, probability sampling could not be implemented.

### 2.8 Statistical analysis

Descriptive statistics were used to summarize sociodemographic and occupational characteristics, with categorical variables reported as frequencies and percentages. The prevalence of the main outcomes was estimated for the overall sample and stratified by sex. Prevalence was defined as the number of affected individuals divided by the total number of participants, corresponding to the proportion of workers reporting the outcome at the time of the survey (21). Confidence intervals (CIs) were calculated using the Agresti–Coull method, a simple and conservative approach that provides better coverage and stability than the Wald method (22).

To compare the prevalence of depressive symptoms, psychological distress, and perceived workplace discrimination between women and men, crude and adjusted prevalence ratios (PRs) were estimated using Poisson regression models with robust variance, along with their corresponding 95% CIs. The adjusted model included age, marital status, company type, job function, shift system, and tenure with the company. These covariates were selected *a priori* based on previous literature and their potential role as confounders in the association between sex and the study outcomes. All analyses were performed using STATA version 18.0 (23). Statistical significance was defined as a *p*-value < 0.05 and a confidence interval that did not include the null value (PR = 1), indicating a statistically significant difference in prevalence between groups. A complete-case analysis was conducted, excluding participants with missing data for any variables included in the regression models. The number of complete cases is reported in the corresponding tables.

## 3 Results

### 3.1 Sample characteristics

Table 1 presents the characteristics of the overall sample stratified by sex. The study included 712 workers from a large-scale mining company in Chile, of whom 79.9% were men and 20.1% were women. The age distribution differed significantly by sex, with women being younger on average and predominantly concentrated in the 30–44-year age group, whereas men were more frequently represented in the ≥45-year age group. Only a small proportion of participants were aged 29 years or younger.

**Table 1:** Sociodemographic and occupational characteristics of the sample, by sex.

Variable	Men (*n* = 569)	Women (*n* = 143)	Total (*n* = 712)	*p*-value
Age				**< 0.001**
Young workers ( ≤ 29 years)	10 (1.7)	7 (4.9)	17 (2.3)	
Mid-aged workers (30–44 years)	261 (45.8)	90 (62.9)	351 (49.3)	
Older workers (≥45 years)	298 (52.3)	46 (32.1)	344 (48.3)	
Company type				0.930
Principal company	404 (71.0)	101 (70.6)	505 (70.9)	
Contractor	165 (29.0)	42 (29.3)	207 (29.0)	
Marital status				**< 0.001**
Single	165 (29.0)	81 (56.6)	246 (34.5)	
Married	335 (58.8)	39 (27.2)	374 (52.5)	
Civil union	11 (1.9)	5 (3.5)	16 (2.2)	
Separated	23 (4.0)	6 (4.2)	29 (4.0)	
Divorced	32 (5.6)	12 (8.3)	44 (6.1)	
Widowed	3 (0.5)	0 (0.0)	3 (0.4)	
Parental status				**< 0.001**
No	68 (11.9)	52 (36.3)	120 (16.8)	
Yes	501 (88.0)	91 (63.6)	592 (83.1)	
Educational level				**< 0.001**
Primary/secondary education	166 (29.1)	9 (6.2)	175 (24.5)	
Technical education	159 (27.9)	27 (18.8)	186 (26.1)	
University (degree/Postgraduate)	244 (42.8)	107 (74.8)	351 (49.3)	
Shift work				**< 0.001**
No	164 (28.8)	78 (54.5)	242 (33.9)	
Yes	405 (71.1)	65 (45.4)	470 (66.0)	
Shift type				**< 0.001**
4 x 3	25 (6.1)	16 (24.6)	41 (8.7)	
4 x 4	247 (61.1)	25 (38.4)	272 (57.9)	
5 x 2	28 (6.9)	3 (4.6)	31 (6.6)	
7 x 7	75 (18.5)	20 (30.7)	95 (20.2)	
6 x 3	19 (4.7)	0 (0.0)	19 (4.0)	
Other	10 (2.4)	1 (1.5)	11 (2.3)	
Job role				**< 0.001**
Administrative	79 (13.8)	48 (33.5)	127 (17.8)	
Fieldwork	298 (52.3)	37 (25.8)	335 (47.0)	
Mixed	192 (33.7)	58 (40.5)	250 (35.1)	
Company tenure				**< 0.001**
< 5 years	270 (47.4)	94 (65.7)	364 (51.1)	
5–10 years	111 (19.5)	26 (18.1)	137 (19.2)	
11–20 years	145 (25.4)	21 (14.6)	166 (23.3)	
>20 years	43 (7.5)	2 (1.4)	45 (6.3)	
Mining tenure				**< 0.001**
< 5 years	62 (10.9)	52 (36.3)	114 (16.0)	
5–10 years	73 (12.8)	30 (20.9)	103 (14.5)	
11–20 years	270 (47.4)	53 (37.0)	323 (45.4)	
>20 years	164 (28.8)	8 (5.5)	172 (24.1)	
Awareness of anti-discrimination policy				0.165
No	99 (17.4)	18 (12.5)	117 (16.4)	
Yes	470 (82.6)	125 (87.4)	595 (83.6)	

Percentages may not total 100% due to rounding. *p*-values correspond to chi-square tests comparing proportions between men and women. “Shift work” refers to rotating shift systems. “Mixed” job roles include both administrative and field tasks. Bold values indicate statistically significant differences between men and women (*p* < 0.05).

Most participants worked for the principal company (70.9%), with no meaningful differences by sex. Significant differences were observed in marital status: marriage was more common among men (58.9%), whereas women were more frequently single (56.6%).

Regarding parental status, 83.2% of participants reported having children, with a higher proportion among men (88.1%) than women (63.6%). Educational attainment also differed by sex: 74.8% of women reported holding a university or postgraduate degree, compared with 42.9% of men.

Overall, 66.0% of workers reported working in rotating shift systems, with a higher prevalence among men (71.2%) than women (45.5%). Among shift workers, the most common schedules were 4 × 4 (58.0%) and 7 × 7 (20.3%), with significant sex differences in shift distribution.

Regarding job functions, most participants performed field-based work (47.1%), followed by mixed roles (35.1%) and administrative positions (17.8%). Men were predominantly engaged in field-based work (52.4%), whereas women were more frequently represented in administrative (33.6%) and mixed roles (40.6%).

Differences were also observed in tenure within both the company and the mining sector. A greater proportion of women had fewer than 5 years of experience in the company (65.7%) and in the mining sector (36.4%), whereas men generally reported longer employment histories.

Finally, 83.6% of workers reported being aware of the company's workplace anti-discrimination policy.

### 3.2 Prevalence of depressive symptoms, psychological distress, and perceived workplace discrimination

As shown in Table 2, the prevalence of depressive symptoms among workers was 8.3% (95% CI: 6.44–10.65). When stratified by sex, women showed a significantly higher prevalence (14.5%) than men (6.8%). Prevalence ratio analyses confirmed this difference, indicating that women were more likely to experience depressive symptoms than men. The crude prevalence ratio was 2.12 (95% CI: 1.26–3.57; *p* = 0.004), indicating that the prevalence of depressive symptoms was more than twice as high among women. This association remained statistically significant after adjustment for covariates, with an adjusted prevalence ratio of 1.81 (95% CI: 1.01–3.25; *p* = 0.046) (Table 3), supporting a statistically significant association between female sex and a higher prevalence of depressive symptoms.

**Table 2:** Prevalence of psychological distress, depression, and perceived workplace discrimination among workers, by sex.

Variable	Men			Women			Total		
Psychological distress	**(*****n*** = **559)**	**%**	**95 % CI**	**(*****n*** = **137)**	**%**	**95 % CI**	**(*****n*** = **696)**	**%**	**95 % CI**
No	482	86.2	83.11–88.85	108	78.8	71.21–84.88	590	84.8	81.90–87.25
Yes	77	13.8	11.15–16.89	29	21.2	15.12–28.79	106	15.2	12.75–18.09
Depression	**(*****n*** = **543)**	**%**	**95 % CI**	**(*****n*** = **131)**	**%**	**95 % CI**	**(*****n*** = **674)**	**%**	**95 % CI**
No	506	93.2	90.73–95.04	112	85.5	78.37–90.60	618	91.7	89.35–93.56
498ptYes	37	6.8	4.96–9.27	19	14.5	9.40–21.62	56	8.3	6.44–10.65
Perceived workplace discrimination	**(*****n*** = **541)**	**%**	**95 % CI**	**(*****n*** = **130)**	**%**	**95 % CI**	**(*****n*** = **671)**	**%**	**95 % CI**
No	474	87.6	84.56–90.14	106	81.5	73.94–87.33	580	86.4	83.63–88.83
498ptYes	67	12.4	9.86–15.44	24	18.5	12.67–26.06	91	13.6	11.17–16.37
Observed workplace discrimination	**(*****n*** = **543)**	**%**	**95 % CI**	**(*****n*** = **131)**	**%**	**95 % CI**	**(*****n*** = **674)**	**%**	**95 % CI**
No	443	81.6	78.10–84.63	101	77.1	69.15–83.50	544	80.7	77.56–83.52
Yes	100	18.4	15.37–21.90	30	22.9	16.50–30.85	130	19.3	16.48–22.44

Prevalence estimates are presented with 95% confidence intervals calculated using the Agresti–Coull method. The analytical sample varied across outcomes because of missing responses (psychological distress: *n* = 696/712, 16 missing; depressive symptoms: *n* = 674/712, 38 missing; perceived workplace discrimination: *n* = 671/712, 41 missing; observed workplace discrimination: *n* = 674/712, 38 missing). “Depression” was defined as a PHQ-9 score ≥10; “psychological distress” as a K6 score ≥7. “Perceived workplace discrimination” refers to self-reported experiences, whereas “observed workplace discrimination” refers to witnessing discriminatory acts toward others.

**Table 3:** Prevalence ratios of depression, psychological distress, and workplace discrimination among workers, by sex.

Variables	Crude prevalence ratio	95 % CI	*p*-value	Adjusted prevalence ratio	95 % CI	*p*-value
Depression (*n* = 674)	2.12	1.26–3.57	**0.004**	1.81	1.01–3.25	**0.046**
Psychological distress (*n* = 696)	1.53	1.04–2.25	**0.028**	1.30	0.85–1.97	0.218
Perceived workplace discrimination (*n* = 671)	1.49	0.97–2.28	0.066	1.67	1.04–2.68	**0.032**
Observed workplace discrimination (*n* = 674)	1.24	0.86–1.78	0.237	1.20	0.82–1.77	0.333

Crude and adjusted prevalence ratios (PRs) were estimated using Poisson regression models with robust variance. The sample size for each model is indicated in parentheses; differences reflect missing data in the corresponding outcome. Adjusted models included age, marital status, company type, job role, shift work, and company tenure. Associations were considered statistically significant when *p* < 0.05 and the 95% CI did not include 1. Bold values indicate statistically significant associations.

Following administration of the PHQ-9, an additional question assessed the level of difficulty participants experienced in carrying out work-related, household, or social responsibilities. As shown in Table 4, most men reported no difficulty performing these activities (70.5%), whereas women more frequently reported functional limitations, with “somewhat difficult” being the most common response (43.2%), followed by “very difficult” (9.6%).

**Table 4:** Difficulty in fulfilling work, household, or social responsibilities (*n* = 599).

Response category	Men (*n* = 474)	Women (*n* = 125)	Total (*n* = 599)
	***N*** **(%)**	***N*** **(%)**	***N*** **(%)**
Not at all difficult	334 (70.5)	59 (47.2)	393 (65.6)
Somewhat difficult	126 (26.6)	54 (43.2)	180 (30.0)
Very difficult	12 (2.5)	12 (9.6)	24 (4.0)
Extremely difficult	2 (0.4)	0 (0.0)	2 (0.3)

This question was administered to participants who completed the PHQ-9 (*n* = 674) to assess functional impairment associated with depressive symptoms. A total of 599 participants answered this question, as it was not mandatory. Therefore, these results are descriptive and should be interpreted accordingly.

The prevalence of psychological distress was 15.2% in the overall sample. Among women, prevalence increased to 21.2%, compared with 13.8% among men (Table 2). As shown in Table 3, the crude prevalence ratio was 1.53 (95% CI: 1.04–2.25; *p* = 0.028), suggesting that women were 53.0% more likely to report psychological distress than men, indicating greater vulnerability in this group. However, this association was no longer statistically significant after adjustment for sociodemographic and occupational covariates.

Table 5 presents workers' attributions regarding the causes of their psychological distress, revealing differences by sex. Whereas 47.8% of men attributed their distress to “other circumstances,” 43.4% of women attributed it to both paid employment and unpaid responsibilities. Paid employment alone was reported in similar proportions among men (23.2%) and women (22.5%).

**Table 5:** Attribution of psychological distress symptoms.

Attribution category	Men (*n* = 439)	Women (*n* = 129)	Total (*n* = 568)
	***N*** **(%)**	***N*** **(%)**	***N*** **(%)**
Paid work	102 (23.2)	29 (22.5)	131 (23.1)
Paid and unpaid work	106 (24.1)	56 (43.4)	162 (28.5)
Unpaid domestic work	21 (4.8)	4 (3.1)	25 (4.4)
Other circumstances	210 (47.8)	40 (31.0)	250 (44.0)

This item was presented only to participants who completed the K6 scale (*n* = 696) to assess functional impairment associated with psychological distress. A total of 568 participants responded to this question, as it was not mandatory. Therefore, these results are descriptive and should be interpreted accordingly.

As shown in Table 2, the prevalence of perceived workplace discrimination was 13.6% in the overall sample. When stratified by sex, women reported a higher prevalence (18.5%) than men (12.4%). This difference is reflected in the crude prevalence ratio of 1.49 (95% CI: 0.97–2.28; *p* = 0.066) (Table 3), which approached statistical significance. After adjustment for covariates, the prevalence ratio increased to 1.67 (95% CI: 1.04–2.68; *p* = 0.032), indicating that the prevalence of perceived workplace discrimination was 67.0% higher among women than among men.

Regarding discrimination witnessed in the workplace, 19.3% of workers reported having observed discriminatory acts directed toward others (Table 2). This proportion was also higher among women (22.9%) than men (18.4%). However, no statistically significant sex differences were identified. The crude prevalence ratio was 1.24 (95% CI: 0.86–1.78; *p* = 0.237), and the adjusted prevalence ratio was 1.20 (95% CI: 0.82–1.77; *p* = 0.333) (Table 3).

The main reasons reported for both perceived and observed discrimination followed similar patterns. The category “other” was the most frequently reported in both cases (51.0 and 34.0%, respectively), mainly comprising reasons related to organizational and working conditions (e.g., subcontracting status, hierarchical position, and employment opportunities), interpersonal relationships in the workplace (e.g., conflicts, exclusion, and personal attitudes), and characteristics of discriminatory events (e.g., perpetrator or type of discrimination). This was followed by educational level, physical appearance, and sex. Discriminatory acts were primarily attributed to coworkers (70.0%) and direct or indirect supervisors (75.0%). These incidents occurred in both formal contexts (53.0%), such as meetings or performance evaluations, and informal contexts (57.0%), such as casual conversations or social interactions.

Regarding frequency, most participants reported that discriminatory acts occurred infrequently: 45.0% indicated that they occurred a few times per year, and 38.0% reported a few times per month. Psychological aggression was the most commonly reported form of discrimination (82.0%), whereas physical aggression was rare, with 96.0% of participants disagreeing or strongly disagreeing that this type of violence occurred. Finally, 74.0% of workers reported that discrimination had negatively affected their health, and 64.0% indicated that it had adversely impacted their work performance.

## 4 Discussion

The aim of this study was to examine sex-related differences in depressive symptoms, psychological distress, and perceived workplace discrimination among workers employed in a large-scale mining company in Chile. The findings revealed moderate prevalences of depressive symptoms (8.3%), psychological distress (15.2%), and perceived workplace discrimination (13.6%). These results should be interpreted within the mining context, which is characterized by high physical and psychological demands, remote operations, demanding work schedules, and an organizational culture that has historically been male dominated (24, 25).

From a human-centered and organizational ergonomics perspective, depressive symptoms, psychological distress, and workplace discrimination can be understood as indicators of how work systems support workers' health, inclusion, and occupational wellbeing. When stratified by sex, women exhibited higher rates across all three outcomes, and differences in depressive symptoms and perceived workplace discrimination remained statistically significant after adjustment for sociodemographic and occupational covariates. Although these associations should be interpreted with caution given the limited precision of some estimates and the possibility of residual confounding, they suggest that psychosocial inequalities may remain present within the mining sector and could represent barriers to sustainable work, particularly in relation to workforce retention, equitable participation, and long-term occupational wellbeing among women in a traditionally male-dominated industry.

### 4.1 Depression

This study found that women had a higher prevalence of depressive symptoms than men, with the adjusted analysis indicating approximately twice the prevalence. Although the estimate was statistically significant, its confidence interval suggests limited precision. This pattern is consistent with both national and international patterns (26, 27). In Chile, the National Health Survey reported prevalences of 21.7% among women and 10.0% among men, corresponding to a prevalence ratio of 2.17 (28). Our findings show a similar gender gap, although the overall prevalence was lower (8.3%), possibly due to protective characteristics of the study population, such as greater job stability and higher income levels, both of which have been associated with a reduced risk of depression (29).

The masculine culture that characterizes the mining sector may also inhibit emotional expression and help-seeking behaviors. Psychological distress is often perceived as a sign of weakness, potentially leading to underreporting of symptoms. Matamala and Barrera (25) argue that masculinity functions as a psychological defense mechanism, reinforcing a culture that values toughness and discourages expressions of vulnerability, with negative consequences at both individual and collective levels. Similarly, Seidler (30) highlights that adherence to traditional masculine norms, such as self-reliance and emotional restriction, may hinder help-seeking behaviors, particularly among men. From an organizational ergonomics perspective, these cultural norms may act as barriers that limit the recognition and management of psychosocial risks within the work system.

Although the prevalence observed in this study was lower than that reported in previous mining-sector research, such as the 23% prevalence reported by Ansoleaga and Toro (10), it remains a concern given the demanding working conditions commonly found in the industry. This discrepancy may reflect advances in occupational health policies and practices, including the implementation of the National Protocol for the Surveillance of Psychosocial Risks at Work (31, 32). International studies have reported substantial variability in the prevalence of depressive symptoms among mining workers (33–37), likely reflecting differences in working conditions, organizational environments, and sociocultural contexts.

### 4.2 Psychological distress

In this study, 15.2% of participants reported high or very high levels of psychological distress. This prevalence is lower than the 34% reported by Ansoleaga (38) among public hospital workers, but is consistent with findings from other Chilean studies, including prevalences of 14.1% among salaried workers (19) and 17.2% among hospital personnel (18). These comparisons are methodologically valid because they rely on the same K6 scale and cutoff point. Despite the demanding nature of mining work, levels of psychological distress appear comparable to those observed in other occupational sectors. This finding suggests that psychological distress may reflect broader structural challenges affecting the Chilean workforce as a whole, reinforcing the need for cross-sector preventive strategies.

Although the mining industry is subject to extensive regulation, its distinctive characteristics, including remote work locations, extended work schedules, high production demands, and rigid organizational cultures, create specific psychosocial risks. The underreporting of psychological distress in male-dominated sectors such as mining may reflect both gender norms and the lack of safe environments for emotional expression. From an organizational ergonomics perspective, these characteristics represent features of the work system that may influence workers' psychological wellbeing and their capacity to participate sustainably in work.

Women reported higher levels of psychological distress (21.2%) than men (13.8%), consistent with previous research (18, 19, 38). However, this difference was no longer statistically significant after adjustment for occupational and sociodemographic variables, suggesting that occupational and organizational conditions may play a more important role than biological sex alone in shaping psychological distress (39). These findings highlight the importance of examining how sex and gender interact with occupational determinants to shape mental health outcomes. In the mining industry, where women and men often follow different career trajectories and occupy different positions within the workforce, such analyses are essential for understanding mental health inequalities.

International evidence shows considerable variability in levels of psychological distress among mining workers across different countries and settings (40–43), suggesting that organizational, occupational, and contextual factors may play an important role.

### 4.3 Workplace discrimination

The prevalence of perceived workplace discrimination (13.6%) and observed workplace discrimination (19.3%) identified in this study was moderate but nonetheless meaningful, particularly considering the potential for underreporting due to stigma, normalization of discriminatory behaviors, or fear of retaliation. National evidence suggests a higher prevalence of workplace discrimination, with one study reporting a rate of 26.4% (44). However, methodological differences limit direct comparisons between studies.

Nevertheless, these comparisons remain relevant because they indicate that workplace discrimination in Chile is neither uncommon nor isolated. Other studies have also identified the workplace as a key setting for perceived discrimination, reporting prevalence estimates ranging from 40 to 46%, with coworkers being the most frequently identified perpetrators (45, 46). Consistent with these findings, more than 70% of participants in our study attributed discriminatory acts to coworkers, while 75% identified direct or indirect supervisors as responsible.

The lower prevalence observed in the present study may reflect stronger organizational practices or more precise measurement approaches. However, it may also underestimate the true prevalence of workplace discrimination due to institutional silencing, ambiguity in the definition of discriminatory behaviors, or the absence of safe and effective reporting mechanisms. Despite these limitations, the findings reinforce the notion that the workplace remains a setting where tensions and inequalities persist, particularly in male-dominated industries. From a human-centered ergonomics perspective, workplace discrimination may be interpreted as an indicator of deficiencies in the social and organizational dimensions of the work system, affecting workers' sense of inclusion, psychological safety, and occupational wellbeing.

Sex-disaggregated analyses revealed a persistent gender gap. Observed discrimination was more frequently reported by women than by men, although this difference did not reach statistical significance. Previous research suggests that gender socialization processes and greater awareness of inequality may contribute to women's increased ability to recognize and report discriminatory experiences (47).

Similarly, perceived workplace discrimination was more prevalent among women. Although this difference was not statistically significant in the crude analysis, it became significant after adjustment for company type, job role, shift system, marital status, and job tenure. These findings indicate that sex was significantly associated with perceived workplace discrimination in this study. This association may be particularly relevant in contexts such as mining, which are characterized by rigid hierarchies, masculine organizational cultures, and limited female representation. In such environments, discriminatory practices may become normalized or embedded within informal workplace dynamics, making them more difficult to identify and address. This highlights the importance of understanding how organizational conditions shape workers' experiences and contribute to inequalities in workplace inclusion and occupational wellbeing.

The prevalence of approximately 13% observed in this study is lower than the 27.6% reported among workers in an Australian mining company, where employees who experienced workplace discrimination were nearly five times more likely to report depression and almost three times more likely to report anxiety (40). Furthermore, the Rio Tinto organizational culture report found that 48.4% of employees had experienced bullying or harassment and 11.7% had experienced racism, with women and Indigenous workers being the most affected groups. These results were based on data collected from employees across 35 countries (5).

### 4.4 Structural barriers and segregation in the mining sector

One of the main findings of this study is the existence of disparities between women and men in mental health outcomes and workplace discrimination. Women reported a higher prevalence of depressive symptoms, psychological distress, and perceived workplace discrimination, with two of these outcomes remaining statistically significant after adjustment. Although previous studies have consistently shown that women experience higher rates of depression and psychological distress in the general population (26, 27, 48–50), our findings suggest that these inequalities may be amplified in male-dominated work environments.

These differences persisted despite the presence of several potentially protective factors. Most women in the sample were younger, single, and had higher levels of educational attainment, yet they reported shorter job tenure and limited participation in field-based roles. In contrast, men had lower educational attainment, longer job tenure, and were predominantly employed in operational field positions. These patterns suggest that structural barriers continue to restrict women's access to, retention in, and advancement within the mining industry. From an organizational ergonomics perspective, these patterns may reflect systemic characteristics of the work environment that influence opportunities for participation, career progression, and long-term workforce integration.

Despite their higher educational attainment, women remained concentrated in administrative and mixed roles, whereas men predominated in operational positions. The relatively low proportion of mothers among female participants may reflect decisions to postpone or forgo motherhood in anticipation of workplace barriers or due to inadequate working conditions for balancing family and professional responsibilities. Similarly, women's shorter job tenure may indicate processes of exclusion or challenges related to workforce retention. A recent study conducted in Indonesia identified a comparable pattern, in which highly qualified women working in the mining sector reported anxiety and stress associated with excessive workloads and insufficient professional recognition (51).

The observed sex differences in mental health outcomes and workplace discrimination may also have implications for sustainable work in the mining sector. Sustainable work refers to the capacity of work systems to support workers' health, wellbeing, and continued participation throughout their working lives. From this perspective, persistent inequalities in psychosocial health and workplace experiences may undermine retention, career development, and long-term workforce participation among women, potentially limiting efforts to build a more diverse and sustainable mining workforce.

### 4.5 Double burden, functional limitations, and mental load

Gender differences were also evident in the functional limitations reported by participants. Men reported fewer difficulties balancing their various roles and were less likely than women to attribute psychological distress to the combined demands of paid work and unpaid domestic responsibilities. These findings highlight the unequal distribution of domestic and emotional labor and underscore the impact of the double burden on women's wellbeing.

In Chile, women spend more than two additional hours per day on unpaid domestic work compared with men (52). This unequal distribution reflects not only household responsibilities but also deeply rooted social expectations regarding gender roles. The resulting mental load may contribute to emotional exhaustion, psychological distress, and reduced work performance.

Addressing these disparities requires more than establishing numerical targets for female workforce participation. Although Chile's National Mining Policy 2050 has established inclusion goals (12), meaningful progress will depend on broader cultural, institutional, and regulatory transformations. As Tynan et al. (53) have argued, gender-responsive mental health strategies should support both women and men by recognizing the intersection of occupational demands, domestic responsibilities, and gender norms.

These findings reinforce the importance of considering psychosocial health as a key dimension of occupational wellbeing. Efforts to improve mental health in the mining sector should therefore extend beyond individual-level interventions and address the broader social and organizational conditions that shape workers' experiences both inside and outside the workplace.

### 4.6 Workplace discrimination: a latent psychosocial risk

Workplace discrimination should not be viewed solely as an interpersonal issue but rather as a psychosocial risk with structural implications, particularly in sectors such as large-scale mining. Although its prevalence in this study appears relatively low, its higher frequency among women warrants urgent attention. Previous research has shown that workplace discrimination adversely affects mental health and job performance, contributing to anxiety, emotional exhaustion, sleep and mood disorders, demotivation, reduced concentration, and work disengagement (54, 55).

Furthermore, it undermines perceptions of organizational justice, deteriorates the work environment, and erodes employees' sense of belonging. Consistent with this literature, participants who reported experiencing workplace discrimination also reported perceived negative impacts on their wellbeing and work performance, reinforcing existing inequalities and increasing psychosocial vulnerability in the workplace.

From an organizational ergonomics perspective, workplace discrimination can be understood not only as an interpersonal phenomenon but also as a characteristic of the work system. Organizational cultures, supervisory practices, communication processes, and power structures may contribute to the emergence, maintenance, or normalization of discriminatory behaviors. Consequently, interventions should extend beyond individual-level approaches and address organizational conditions that influence psychosocial health and workplace inclusion.

A notable finding was the complexity of the reasons reported for discrimination. Although participants were offered several predefined categories, the option “other” was selected most frequently, suggesting the presence of forms of discrimination that extend beyond conventional classifications. Three main themes emerged: (i) interpersonal conflicts; (ii) workers' physical or mental health conditions; and (iii) organizational factors, such as subcontracting status. This diversity highlights the multifaceted nature of workplace discrimination and the need for comprehensive responses.

Other reported reasons included educational attainment, physical appearance, and sex, revealing more subtle forms of exclusion linked to symbolic capital, aesthetic norms, and gender roles (56). In male-dominated environments such as mining, physical appearance and conformity to gendered expectations may influence inclusion and acceptance, while educational attainment may function as an informal status marker that reinforces internal inequalities.

From a psychosocial perspective, workplace discrimination is not merely an individual-level problem but is closely linked to multiple workplace determinants (57). Consequently, it has structural effects and important public health implications. Addressing discrimination on a case-by-case basis is insufficient; comprehensive public policies, gender-responsive strategies, and the promotion of safer, fairer, and more inclusive work environments are needed to effectively address this issue.

### 4.7 Implications for human-centered ergonomics and sustainable work

The present findings have important implications for human-centered ergonomics and the promotion of sustainable work in male-dominated industries such as mining. Human factors and ergonomics adopts a systems-oriented and design-driven perspective aimed at simultaneously optimizing worker wellbeing and system performance (58). Research on psychosocial safety climate (PSC) suggests that organizational conditions influence the occurrence of discrimination, harassment, workplace violence, and other adverse social-relational experiences, while simultaneously shaping workers' psychological health and wellbeing (59). In this sense, discrimination can be understood as a psychosocial risk embedded within the organizational context, reflecting how work environments are designed, managed, and experienced by workers. This interpretation is consistent with evidence indicating that psychosocial working conditions are modifiable through organizational-level interventions and that improvements in the psychosocial work environment can contribute to better health, wellbeing, and long-term workforce participation (60). Therefore, addressing discriminatory organizational practices may represent an important pathway toward creating healthier and more inclusive work environments.

Beyond its implications for psychosocial wellbeing, the co-occurrence of workplace discrimination and adverse mental health outcomes observed in this study suggests that creating sustainable work systems requires more than controlling physical hazards; it also demands organizational conditions that foster inclusion, psychological safety, and equitable participation (59, 61). Human-centered approaches emphasize designing work environments that accommodate workforce diversity and support workers' health, capabilities, and participation throughout their working lives (61). This issue may be particularly relevant in male-dominated sectors such as mining, where women remain underrepresented and may encounter structural barriers that limit inclusion, participation, and career development. In this context, promoting gender-inclusive organizational cultures may contribute not only to improved mental health but also to workforce retention, work ability, long-term employability, and sustainable workforce participation (62). These findings reinforce the need to integrate psychosocial and equity considerations into occupational health and ergonomics strategies, advancing sustainable, healthy, and inclusive workplaces.

### 4.8 Limitations and strengths

This study has several limitations. First, different sources of bias may have influenced the findings. Social desirability bias may have led to underreporting of the outcomes assessed, recall bias may have affected responses given the one-year reference period used to evaluate workplace discrimination, and selection bias is also possible, as participation was voluntary and the overall response rate could not be calculated because the total number of eligible contractor workers was unavailable. Consequently, workers with greater interest in mental health or workplace discrimination may have been more likely to participate, potentially affecting the representativeness of the study sample. In addition, the cross-sectional design precludes causal inference regarding the associations observed in this study. The possibility of a healthy worker survivor effect should also be considered, as workers experiencing greater psychosocial burden may have been more likely to leave the mining industry before the study was conducted, potentially leading to an underestimation of the prevalence of adverse psychosocial outcomes. Although the analyses were adjusted for relevant covariates, residual confounding cannot be ruled out.

The workplace discrimination scale used in this study was developed and validated within the Chilean context. While this enhances its contextual relevance, it limits international comparability due to the absence of external validation. Nevertheless, the instrument allowed the assessment of context-specific dimensions of discrimination that are often overlooked by standardized measures. Furthermore, all study variables were obtained through self-reported questionnaires. Therefore, the possibility of common-method bias cannot be excluded. In addition, no objective organizational or clinical data were available to complement the self-reported information.

The findings are based on data from a single large-scale mining company, which may limit their generalizability to other mining companies, smaller mining operations, or mining sectors in other countries. However, the sample included a broad range of occupational roles and a proportion of women (20%) that exceeds the national average for the mining industry, allowing for meaningful sex-stratified analyses.

A key strength of this study is its integrated assessment of depressive symptoms, psychological distress, and perceived workplace discrimination using validated instruments. This approach provides a comprehensive perspective on psychosocial risks in the large-scale mining sector. In addition, the study contributes evidence relevant to ongoing discussions regarding occupational wellbeing, workplace inclusion, and sustainable work in male-dominated industries. The findings also provide insights into the potential role of organizational and psychosocial characteristics of work systems in shaping workers' health, inclusion, and long-term participation in the workforce.

### 4.9 Future research directions

Future research should further examine the impact of perceived workplace discrimination on mental health outcomes, taking into account potential mediating factors such as social support networks and organizational culture. Longitudinal studies are needed to establish causal relationships between working conditions and mental health outcomes.

In addition, future studies should evaluate the effectiveness of organizational and human-centered interventions aimed at preventing and mitigating workplace discrimination in male-dominated sectors such as mining. In this regard, an intersectional approach may help elucidate how gender, age, and educational attainment interact to shape experiences of discrimination and mental health.

Future research should also explore how human-centered and organizational ergonomics interventions influence psychosocial outcomes, workplace inclusion, and sustainable workforce participation in the mining sector.

Finally, occupational health policies should consider the specific characteristics of the Chilean mining sector by incorporating locally generated evidence while promoting international comparisons. Such efforts may contribute to the development of more inclusive and effective regulatory frameworks for protecting mental health and advancing equity in the workplace.

## 5 Conclusions

This study examined sex-related differences in depressive symptoms, psychological distress, and perceived workplace discrimination among workers employed in a large-scale mining company in Chile, showing that women experienced a greater psychosocial burden than men. Women reported higher prevalences across all three outcomes, and after adjustment for sociodemographic and occupational characteristics, sex differences remained statistically significant for depressive symptoms and perceived workplace discrimination. These findings indicate associations rather than causal relationships and suggest the presence of sex-based disparities in psychosocial health and workplace experiences within this mining workforce.

The findings highlight the importance of addressing psychosocial risks and workplace discrimination as key occupational health priorities. From an organizational ergonomics and human-centered perspective, workplace strategies that promote inclusion, psychological safety, and equitable working conditions may contribute to healthier and more sustainable work environments.

## Data Availability

The datasets presented in this article are not readily available because the datasets generated and analyzed during the current study are not publicly available because they contain confidential information obtained under a confidentiality agreement with the participating mining company. Requests for access to de-identified data may be considered by the corresponding author on a case-by-case basis, subject to approval by the participating company and compliance with the applicable confidentiality agreements and ethical requirements. Requests to access the datasets should be directed to ignacio.castellucci@uv.cl.
